# Influence of High Temperature and Ammonia and Nitrite Accumulation on the Physiological, Structural, and Genetic Aspects of the Biology of Largemouth Bass (*Micropterus salmoides*)

**DOI:** 10.3390/antiox14040495

**Published:** 2025-04-20

**Authors:** Yuexing Zhang, Hui Qiao, Leyang Peng, Yujie Meng, Guili Song, Cheng Luo, Yong Long

**Affiliations:** 1 National Engineering Research Center for Marine Aquaculture, Marine Science and Technology College, Zhejiang Ocean University, Zhoushan 316022, China; yuexing.zhang@zjou.edu.cn (Y.Z.); qiaohui1@zjou.edu.cn (H.Q.); 2 State Key Laboratory of Breeding Biotechnology and Sustainable Aquaculture, Institute of Hydrobiology, Chinese Academy of Sciences, Wuhan 430072, China; pengleyang23@mails.ucas.ac.cn (L.P.); mengyujie@webmail.hzau.edu.cn (Y.M.); guilisong@ihb.ac.cn (G.S.); 3 College of Food Science and Technology, Huazhong Agricultural University, Wuhan 430070, China; 4 Xiaogan Academy of Agricultural Sciences, Xiaogan 432100, China; 15072571717@163.com

**Keywords:** high temperature, ammonia, nitrite, largemouth bass, gene expression

## Abstract

Hyperthermia and nitrogenous pollutants like ammonia and nitrite are common risk factors that adversely affect fish health and pose significant threats to the aquaculture industry. However, the impacts of high temperatures on the accumulation of nitrogenous pollutants in the water of the aquaculture systems and their toxicity to farmed fish are not well understood. In this study, juvenile largemouth bass (*Micropterus salmoides*, LMB) were kept at 28 °C and 34 °C in a closed aquatic system to investigate the effects of higher temperatures on ammonia and nitrite accumulation. The fish were fed 2% of their body weight daily for a 14-day experiment. Ammonia levels gradually increased, peaking on day 7 at 34 °C and on day 9 at 28 °C, then decreased to near zero. Nitrite levels remained low initially and increased rapidly along with the reduction in ammonia levels at both temperatures. The 34 °C high temperature accelerated the accumulation of ammonia and its transformation into nitrite compared to 28 °C. Fish were sampled on day 1 (low ammonia and low nitrite, LALN), day 8 (high ammonia and low nitrite, HALN), and day 14 (low ammonia and high nitrite, LAHN) to explore toxic effects. Successive exposure to high levels of ammonia and nitrite caused oxidative stress in the liver and significant pathogenic changes in the liver and spleen, with more pronounced impacts observed at 34 °C. Significant changes in gene expression were detected in the liver and spleen of fish sampled at HALN and LAHN, compared to those at LALN, with upregulated genes primarily associated with extracellular matrix (ECM) and cytoskeleton organization. A second experiment was conducted at the same temperatures but without ammonia/nitrite accumulation. The results of this experiment confirmed the combined effects of hyperthermia and ammonia/nitrite toxicity on the expression of genes involved in ECM–receptor interaction and TGF-beta signaling. These findings are valuable for optimizing cultivation environments and promoting the health of farmed LMB.

## 1. Introduction

As global temperatures rise, extreme heat events are becoming more frequent and intense [1]. Heat stress is a key factor limiting the development of the aquaculture industry [2,3]. Previous studies have shown that exposure of fish to heat stresses causes the production of reactive oxygen species, leading to oxidative damage to biomolecules and inflammatory reactions [4,5,6]. Heat exposure not only impairs physiological functions [7], growth rate [8], immune function [9,10], and reproductive performance [11] of fish, but also increases susceptibility to diseases [12].

Ammonia is a common pollutant in aquaculture, existing in two forms: ionic ammonia (NH_4_^+^) and nonionic ammonia (NH_3_) in water [13]. The ratio of ionic/nonionic ammonia is influenced by the pH value and temperature of the water [14]. NH_3_, with strong lipid solubility, can easily diffuse into blood and tissues through cell membranes, posing greater toxicity than NH_4_^+^ [15]. Ammonia in aquaculture water mainly originates from animal feces, feed residues, and the metabolism of aquaculture organisms [16]. With the rapid development of intensive aquaculture, there have been frequent occurrences of excessive ammonia (NH_3_ > 0.02 mg/L) in aquaculture water bodies [17,18]. High concentrations of ammonia have significant toxicity to aquatic animals, disrupting their antioxidant systems and inducing oxidative stress [19,20], thereby impairing immune function and increasing disease susceptibility [21,22]. Additionally, ammonia can directly harm vital organs like the liver, spleen, and gills of fish, causing physiological dysfunction and eventual mortality of the organisms [23,24].

Nitrite is another nitrogenous pollutant commonly found in aquaculture. It is an intermediate product of the nitrogen cycle, primarily generated through the nitrification of ammonia [25]. Studies have indicated that nitrite can hinder antioxidant enzyme activity in aquatic animals [26,27] and compromise their immunological functions [28]. Additionally, nitrite can transform hemoglobin in the blood of aquatic animals into methemoglobin, reducing its ability to carry oxygen and causing tissue hypoxia [25]. This can result in detrimental effects such as increased membrane permeability, tissue autolysis, and necrosis [29]. Exposure to high concentrations of nitrite can weaken the immune system, reduce disease resistance [30], and impede the growth and development of fish [31,32].

Farmed fish are often exposed to heat stress, ammonia, and nitrite simultaneously. This joint exposure not only worsens the negative effects of individual stressors but also leads to more severe impacts on fish due to synergistic effects [33,34]. For instance, in silver carp (*Hyphthalmichthys molitrix*) exposed to a combination of ammonia and nitrite (15 mg/L + 10 mg/L), a synergistic effect was observed on the levels of inflammatory cytokines TNF-α and IL-1β, as well as the integrated biomarker response [35]. Nile tilapia (*Oreochromis nilotica*) exposed to high temperature (32 °C) and ammonia (5 mg/L) showed exacerbated oxidative stress and immune suppression compared to single exposures, resulting in more severe inflammatory reactions and tissue damage [36]. Similarly, joint exposure of spotted sea bass (*Lateolabrax maculatus*) to high temperature (33 °C) and nitrite (16 mg/L) significantly increased the expression of apoptosis-related genes compared to exposure to either high temperature or nitrite alone [37].

Previous studies have mainly focused on the acute toxic effects of high concentrations of ammonia and nitrite on fish by adding nitrogen-containing compounds to the water [38,39,40,41]. However, the chronic toxic effects of environmental levels of these compounds on fish metabolism and immune responses remain poorly understood. In aquaculture, ammonia is primarily generated through microbial decomposition of residual feed, feces, and fish metabolism [42], while nitrite is mainly produced from ammonia nitrification and denitrification [43]. These processes are influenced by various environmental factors, with temperature playing a crucial role. High temperatures can accelerate microbial metabolism, leading to increased organic matter decomposition and the accumulation of ammonia and nitrite, thereby enhancing their toxicity [36,44]. However, there is still a lack of comprehensive understanding of how high temperatures impact the production, accumulation, and transformation of ammonia and nitrite in aquaculture water, as well as the impacts of elevated temperatures on the toxicity of these nitrogen-containing pollutants.

Largemouth bass (*Micropterus salmoides*, LMB) is a highly valued species in aquaculture due to its economic significance. However, studies have shown that LMB is vulnerable to heat stress [45,46]. Exposure to temperatures above 33 °C triggers endoplasmic reticulum stress and cell apoptosis in the liver of LMB [46]. Prolonged exposure to 35 °C can completely halt LMB growth [47], and temperatures exceeding 36 °C result in high mortality rates in LMB [48]. Although the effects of heat exposure on the immunological functions of LMB have been studied [12], the impacts of heat stress on the toxicity of nitrogenous pollutants such as ammonia and nitrite remain unclear. This study aims to investigate the impact of a high temperature of 34 °C on the accumulation of nitrogenous pollutants and their toxicity to the liver and spleen of LMB. Our findings are valuable for optimizing breeding environments and promoting the healthy and sustainable development of the LMB aquaculture industry.

## 2. Materials and Methods

### 2.1. Chemicals and Reagents

Paraformaldehyde (PFA) and MS-222 were obtained from Sigma Aldrich (Saint Louis, MO, USA). General chemicals such as alcohol, paraffin, xylene, ammonium chloride, potassium sodium tartrate, sulfanilamide sodium nitrite, potassium nitrate, and naphthalene ethylenediamine hydrochloride were purchased from Acmec Biochemical (Shanghai, China), Macklin (Shanghai, China), or Sinopharm Chemical Reagent Co., Ltd. (Shanghai, China). Nessler’s reagent was obtained from Tianjin Jinbei Fine Chemical Co., Ltd. (Tianjin, China).

### 2.2. Experimental Fish

Largemouth bass fingerlings were obtained from a fish farm in Xiaogan, China. They were transported to the laboratory and raised in indoor round aquaria (height 1.25 m, diameter 1 m) supplied with recycled water. The water temperature was maintained at 28 ± 1 °C, and chemical parameters such as total ammonia nitrogen (<0.1 mg/L), nitrite nitrogen (<0.05 mg/L), and dissolved oxygen (DO, >5 mg/L) were monitored throughout the fish cultivation. The fish room was naturally illuminated, and a commercial extruded feed purchased from Fujian Tianma Science and Technology Group Co., Ltd. (Fuzhou, China) was used for feeding. The nominal feed composition included crude protein (53%), crude lipid (5%), crude fiber (3%), and crude ash (15%). The fish were fed twice daily at 9:00 AM and 3:00 PM until satiated. The animal protocol for this study was approved by the Institutional Animal Care and Use Committee of the Institute of Hydrobiology (Approval ID: E255050101).

### 2.3. Fish Treatment and Sampling

To investigate the effects of high temperatures on the accumulation of ammonia and nitrite in an enclosed aquatic system and their toxicity to largemouth bass, fish were maintained at either 28 °C or 34 °C in aquaria without water exchange and were fed regularly (Experiment #1). A total of 180 individuals with an average body weight of 21.11 ± 0.07 g and an average standard length of 9.48 ± 0.69 cm were divided into six groups of 30 fish each. They were placed in round plastic aquaria with a height of 1.25 m and a diameter of 1 m, filled with 500 L of tap water preconditioned to either 28 °C or 34 °C (3 aquaria for each temperature). The total weight of fish in each group was carefully controlled to ensure an approximately equal amount of feed for the fish and a consistent rate of nitrogenous pollutant accumulation. Water temperature was controlled using aquarium heaters connected to real-time temperature controllers, as previously described [12]. The water was continuously aerated with air stones connected to an air pump to ensure sufficient oxygen supply. Clean water preconditioned to the same temperature was occasionally added to the aquaria to compensate for the volume lost due to evaporation. The fish were fed twice daily at 9:00 AM and 3:00 PM, with a feeding rate of 2% of their body weight. Feces suction and water exchange were not conducted to allow for the accumulation of ammonia and nitrite. To maintain a consistent accumulation rate of nitrogenous pollutants, dead fish were removed and replaced with new fish from the same batch, marked by tail fin clipping. The marked fish were excluded from subsequent analysis.

The experiment lasted for 14 days, and fish were sampled on days 1, 8, and 14 after the start of the experiment. These sampling days were selected based on trends of nitrogenous pollutant accumulation observed in pilot experiments and daily water parameter measurements. This ensured that fish were sampled under specific conditions: low ammonia and low nitrite levels (LALN, day 1), high ammonia and low nitrite levels (HALN, day 8), and low ammonia and high nitrite levels (LAHN, day 14). Two fish were quickly removed from each aquarium for tissue sampling at each time point. The fish were euthanized by immersion in an MS-222 solution (pH 8.0, 250 mg/L) and then dissected. Liver and spleen tissues were collected for further analysis. Fish sampling was conducted in the morning before feeding and lasted no longer than 2 h.

A second experiment (#2) was conducted to study the effects of hyperthermia alone. Ten LMB juveniles (average body weight 64.25 ± 20.13 g, average standard length 9.48 ± 0.69 cm) were randomly assigned to each aquarium. The fish were maintained at either 28 °C or 34 °C (three replicates each), as described above. Waste excretion and water exchange were carried out to prevent the accumulation of ammonia and nitrite. Approximately one-third of the old water was replaced daily with clean water preconditioned to the same temperature. The experiment also lasted for 14 days, and the fish were sampled on days 1, 8, and 14. Two fish were sampled from each aquarium at each time point as well.

### 2.4. Calculation of Growth Rate and Feed Conversion Ratio

The total weight of the fish in each aquarium was measured at the beginning and end of the experiment. The average initial and final weights of the fish in each aquarium were calculated. The feed amount was adjusted after each sampling day by subtracting the weight of the sampled fish from the initial total weight of fish in each aquarium while maintaining a feeding rate of 2%. The total feed consumed by the fish in each aquarium was calculated. Average weight gain rate (WGR, %), average specific growth rate (SGR, %), and feed conversion ratio (FCR) were calculated using the following formulas:

WGR = (Wt − W0)/W0 × 100

SGR = [In(Wt) − In(W0)]/t × 100

FCR = FI/(Wt − W0)

W0 is the average initial weight (g); Wt is the average final weight (g); t is the duration of the experiment (d); FI is the total feed consumed (g).

### 2.5. Water Quality Measurement

Dissolved oxygen and pH in the water were measured using an LDO101 Laboratory Luminescent/Optical Dissolved Oxygen (DO) Sensor (HACH, Loveland, CO, USA) and a FiveEasy Plus pH meter from METTLER TOLEDO (Columbus, OH, USA), respectively. The concentrations of nitrogenous pollutants, including TAN, nitrite, nitrate, and total nitrogen (TN), were determined using the standard methods [49]. Water samples were collected from each aquarium every morning before feeding. Total ammonia–nitrogen (TAN, mg/L) was measured using Nessler’s reagent colorimetry. Nonionic ammonia (NH_3_, mg/L) was calculated as described previously [50]. Nitrite (NO_2_^−^-N, mg/L) was determined using the Griess assay. Nitrate (NO_3_^−^-N, mg/L) was measured using ultraviolet spectrophotometry after removing nitrite with sulfamic acid. Total nitrogen (TN, mg/L) was measured using ultraviolet spectrophotometry after alkaline potassium persulfate digestion of nitrogen compounds into nitrate.

### 2.6. Tisue Biochemical Assay

The freshly collected liver samples were weighed and snap-frozen in liquid nitrogen, then stored at −80 °C. The frozen samples were homogenized with 9-fold volumes of pre-cooled 0.85% NaCl solution for tissue biochemical assays. After centrifugation at 4 °C, 3000 rpm for 10 min, the supernatant was collected for subsequent assays. Protein concentration was measured using the Enhanced BCA Protein Assay Kit from Beyotime (Shanghai, China). The levels of ammonia (#A086-1-1) and malondialdehyde (MDA, #A003-1-1), as well as activities of superoxide dismutase (SOD, #A001-1-1) and catalase (CAT, #A007-1-1), were determined using analytical kits from Nanjing Jiancheng Institute of Biological Engineering (Nanjing, China). These indices were normalized to the protein contents of the samples.

### 2.7. Histological Analysis and TUNEL Assay

Histological analysis and TUNEL (Terminal deoxynucleotidyl transferase (TdT) dUTP Nick-End Labeling) assay were performed following established protocols [51]. Liver and spleen samples were fixed in 4% PFA at 4 °C overnight, dehydrated in alcohol gradients, embedded in paraffin, and sectioned into 4 µm slices. The tissue sections were dewaxed in xylene, rehydrated, and stained with H&E (Hematoxylin and eosin). Slide images were captured using an Aperio VERSA Brightfield, Fluorescence & FISH Digital Pathology Scanner from Leica (Wetzlar, Germany). The same set of slides was used for the TUNEL assay with the One-Step TUNEL Apoptosis Assay Kit from Beyotime (Shanghai, China). Images of the sections were acquired using a Leica SP8 confocal microscope (Wetzlar, Germany). Apoptosis quantification was performed using ImageJ2 (v1.54) [52].

### 2.8. Total RNA Isolation

Two fish were sampled from each aquarium, and their liver and spleen tissues were collected. Total RNA extraction was conducted using the TRIzol reagent from Thermo Fisher Scientific (Waltham, MA, USA) following the manufacturer’s instructions. The integrity of the RNA samples was assessed through agarose gel electrophoresis. The concentrations of the RNA samples were measured using a Q5000 UV-Vis spectrophotometer from Quawell (Sunnyvale, CA, USA).

### 2.9. RNA-Seq and Data Analysis

Total RNA samples extracted from the liver and spleen tissues of the fish in Experiment #1 were subjected to RNA-seq analysis. Six replicates were included for the liver and spleen of each group (28 °C and 34 °C) at each sampling point (1 d, 8 d, and 14 d). In total, 72 libraries were constructed and sequenced for 150 bp at both ends (paired-end 150 bp) using a DNBSEQ-T7 platform from MGI Tech (Shenzhen, China). The raw RNA-seq data generated by this study were deposited in the NCBI Sequence Read Archive (SRA) under the BioProject accession number PRJNA1224590.

The raw RNA-seq data were processed using fastp (v0.23.2) to trim adapters and filter low-quality reads [53]. An index was built for the transcriptome of LMB (NCBI RefSeq assembly GCF_014851395.1), and the clean reads of each sample were used for gene expression quantification with Salmon (v1.10.0) [54]. Gene transcriptional abundance was calculated as TPM (transcripts per million). Genes with a TPM value ≥ 1 in all samples for at least one experimental group were considered expressed. Principal component analysis (PCA) was performed on the gene abundance data using ArrayTrack [55]. Differentially expressed genes (foldchange > 1.5, adjusted *p*-value (*padj*) < 0.05) between the experimental groups were identified using DESeq2 (v1.46.0) [56].

Gene ontology (GO) annotations for the LMB genes were generated using the Pannzer2 web server [57]. Gene ontology enrichment analysis for the gene list of interest was performed with BiNGO (v3.0.5) [58]. The REVIGO (v1.8.1) web server was utilized to eliminate redundant GO terms [59]. Kyoto Encyclopedia of Genes and Genomes (KEGG) pathway enrichment analysis was carried out with clusterProfiler (v4.0) [60]. All genes expressed in the liver or spleen were used as the reference for the functional enrichment analyses. Weighted gene co-expression network analysis was separately conducted on differentially expressed genes in the liver and spleen using WGCNA (v1.73) [61] to identify gene modules and hub genes affected by hyperthermia in response to ammonia/nitrite toxicity. Default parameters were used for WGCNA, with the exception of the soft-thresholding power set to 10, mergeCutHeight set to 0.2, and minModuleSize set to 20. The correlation of gene modules with experimental factors such as temperature, exposure time, and concentrations of ammonia and nitrite in the water was also analyzed. The subnetworks of the gene modules of interest were analyzed using cytoHubba to identify hub genes based on their Maximal Clique Centrality (MCC) scores [62].

### 2.10. Quantitative Real-Time PCR

Quantitative real-time PCR was conducted using a CFX Duet Real-Time PCR System from Bio-Rad (Hercules, CA, USA), as described previously [51]. The EasyScript One-step gDNA Removal and cDNA Synthesis SuperMix from TransGen (Beijing, China) was used for first-strand cDNA synthesis. The target genes, sequences, amplicon sizes, and primer efficiencies are listed in Table S1. To identify stable reference genes for gene expression normalization, we selected candidates (*faua*, *rpl21*, *eif5a*, *rack1*, *rps12*, and *uba52*) based on their consistent transcript levels observed in RNA-seq data. The expression levels of these genes were assessed by qPCR, and their stability was evaluated using NormFinder (version for R, updated in June 2014) (Figure S2) [63]. The geometric mean of *rpl21* and *faua* expression levels was used as the normalization factor for qPCR data analysis.

### 2.11. Statistics

The data are presented as mean ± standard deviation (SD) unless otherwise specified. Statistical analyses were conducted using IBM SPSS Statistics (v25) (Armonk, NY, USA). Differences between two groups were analyzed using Independent Samples *t*-Test, while differences among three or more groups were analyzed using one-way ANOVA followed by multiple comparisons (Duncan). The significance level for the statistical analyses was set at *p* ≤ 0.05. Data were transformed using natural logarithm before statistical analysis if homogeneity of variances was not observed among the experimental groups.

## 3. Results

### 3.1. Feeding-Based Accumulation of Nitrogenous Pollutants

**Figure 1:**
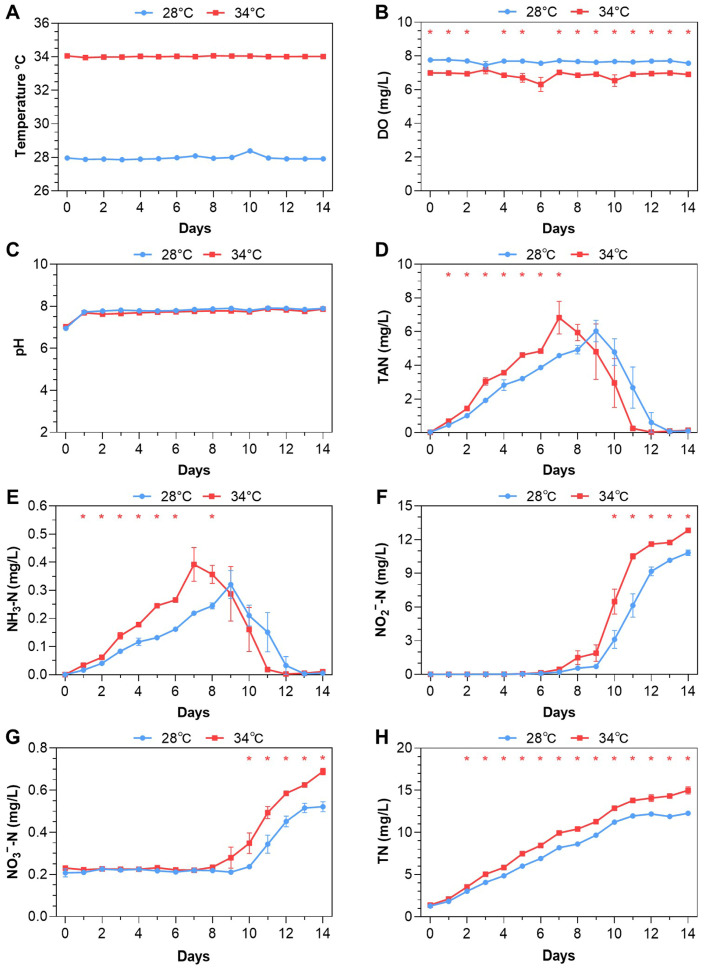
Daily water parameters in Experiment #1: (**A**) Temperature. (**B**) Dissolved oxygen (DO). (**C**) pH value. (**D**) Total ammonia nitrogen (TAN). (**E**) Unionized ammonia (NH_3_-N). (**F**) Nitrite (NO_2_^−^-N). (**G**) Nitrate (NO_3_^−^-N). (**H**) Total nitrogen (TN). The error bars represent standard error (n = 3). *, *p* < 0.05 (independent samples *t*-test).

The experiment (#1) consistently maintained desired temperatures of 28 °C or 34 °C (Figure 1A). Dissolved oxygen (DO) levels at 34 °C were significantly lower (*p* < 0.05) than at 28 °C but remained above 6 mg/L (Figure 1B), suggesting that the fish were not stressed by hypoxia. No significant difference in pH values was identified between 28 °C and 34 °C (Figure 1C).

The concentrations of total ammonia nitrogen (TAN), unionized ammonia (NH_3_-N), and nitrite (NO_2_^−^-N) were initially very low at the start of the experiment (Figure 1D,F). TAN levels gradually increased and peaked on day 7 (6.83 ± 1.68 mg/L) at 34 °C and on day 9 at 28 °C (6.04 ± 1.09 mg/L). Subsequently, TAN levels rapidly decreased to near zero on day 10 at 34 °C and on day 13 at 28 °C. TAN concentrations at 34 °C were consistently higher than those at 28 °C from day 1 to day 7 (*p* < 0.05, Figure 1D). The same variation patterns were observed for unionized ammonia (Figure 1E). Nitrite (NO_2_^−^-N) levels remained below 0.2 mg/L at both temperatures from day 0 to day 6, slowly increased from day 7 to day 9, followed by a rapid rise from day 9 to day 11 at 34 °C or day 12 at 28 °C. The levels continued to rise at a slower rate by the end of the experiment. Nitrite levels at 34 °C were significantly higher than those at 28 °C from day 10 to day 14 (Figure 1F). Nitrate (NO_3_^−^-N) levels remained consistently low (around 0.2 mg/L) from day 0 to day 8, gradually increasing from day 9 at both temperatures. The 34 °C group had significantly higher nitrate levels than the 28 °C group from day 10 to day 14 (Figure 1G). Total nitrogen (TN) levels in both groups increased throughout the experiment, with the 34 °C group showing significantly higher TN levels (*p* < 0.05, Figure 1H). Sampling of fish was conducted on days 1, 8, and 14.

The parameters, including temperature and dissolved oxygen concentration, are nearly the same in Experiment #1 and Experiment #2. Moreover, as expected, the concentrations of total ammonia nitrogen (TAN), ammonia nitrogen (NH_3_-N), and nitrite (NO_2_^−^-N) are very low in Experiment #2 (Figure S1). The concentrations of nitrogenous pollutants measured on the sampling days in both experiments are presented in Table 1.

In summary, we developed a chronic model of consecutive exposure to ammonia and nitrite through feeding and studied the dynamics of ammonia accumulation and conversion to nitrite at two distinct temperatures. A higher temperature of 34 °C initially led to increased ammonia accumulation, followed by elevated nitrite levels in the water compared to 28 °C.

### 3.2. Growth Rate and Feed Conversion Ratio of the Fish

While small mortality occurred at 34 °C, no fish died at 28 °C during the experiment (Table 2). The fish had significantly lower average final weight, average weight gain rate (WGR), and special growth rate (SGR) but significantly higher feed conversion ratio (FCR) at 34 °C than at 28 °C (*p* < 0.05, Table 2).

### 3.3. Ammonia Content and Oxidative Indices in the Liver

**Figure 2:**
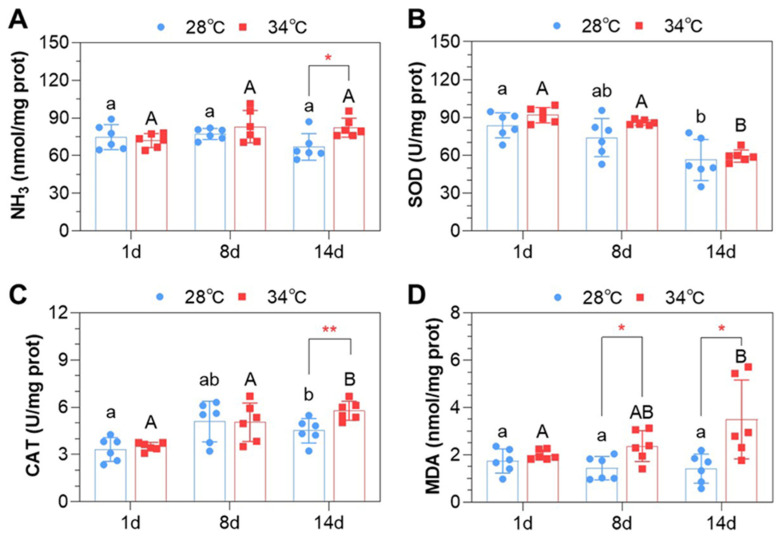
Ammonia content and oxidative parameters of the liver: (**A**) Tissue ammonia content. (**B**) Superoxide dismutase (SOD) activity. (**C**) Catalase (CAT) activity. (**D**) Malondialdehyde (MDA) content. The error bars represent the standard deviation (n = 6). Different sampling days correspond to varying ammonia and nitrite levels: 1 d for low ammonia and low nitrite levels (LALN), 8 d for high ammonia and low nitrite levels (HALN), and 14 d for low ammonia and high nitrite levels (LAHN). Each dot represents one sample. Different lowercase and uppercase letters indicate a significant difference between samples of the same experimental group (28 °C or 34 °C, Duncan multiple comparison test, *p* < 0.05). Significant differences between experimental groups at the same sampling time are indicated as follows: *, *p* < 0.05; **, *p* < 0.01 (independent samples *t*-test).

The fish liver had a significantly higher ammonia content at 34 °C at the end of the experiment (*p* < 0.05, Figure 2A). The SOD activity in the liver decreased in both experimental groups during the experiment, but no significant difference was identified between 28 °C and 34 °C (Figure 2B). The CAT activity increased in both experimental groups from day 1 to day 14. Fish at 34 °C exhibited a significantly higher CAT activity compared to those at 28 °C on day 14 (*p* < 0.01, Figure 2C). The MDA content in the liver increased at 34 °C from day 1 to day 14, while no significant changes were observed at 28 °C. Fish kept at 34 °C showed significantly higher liver MDA contents compared to those at 28 °C on day 8 and day 14 (*p* < 0.05, Figure 2D). In summary, the fish successively exposed to high levels of ammonia (HALN) and nitrite (LAHN) exhibited significant changes in the activities of antioxidant enzymes at both temperatures, with a more pronounced effect observed at 34 °C.

### 3.4. Tissue Damage and Cell Apoptosis

**Figure 3:**
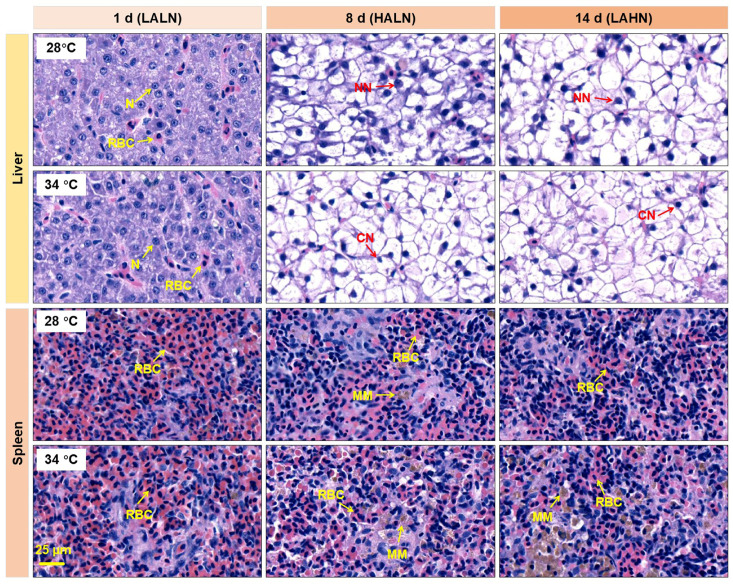
Representative photos of the H&E-stained tissue sections (n = 3). N, nuclear; CN, condensed nuclear; NN, normal nuclear; MM, melanocyte macrophage; RBC, red blood cell. LALN, low ammonia and low nitrite levels; HALN, high ammonia and low nitrite levels; LAHN, low ammonia and high nitrite levels.

The liver sections of fish raised at both temperatures showed nuclear condensation, karyolysis, and swelling of hepatocytes on days 8 and 14, indicating the impacts of exposure to HALN and LAHN (Figure 3). The liver samples from fish raised under both temperatures showed more severe effects on day 14 compared to day 8. Fish raised at 34 °C displayed more pronounced tissue structure changes such as nuclear condensation, karyolysis, and hepatocyte swelling than those raised at 28 °C, suggesting a combined impact of hyperthermia and the toxicities of ammonia and nitrite.

The spleen sections of fish raised at 28 °C on days 8 and 14 showed decreased red blood cell densities, melanocyte macrophage infiltration, and lesion plaques with karyolysis (Figure 3). Similar changes were observed in the spleen sections of fish maintained at 34 °C. In addition, the fish raised at 34 °C had significantly higher numbers of free melanocyte macrophages in the spleen compared to those raised at 28 °C (*p* < 0.05, Figure S3A).

**Figure 4:**
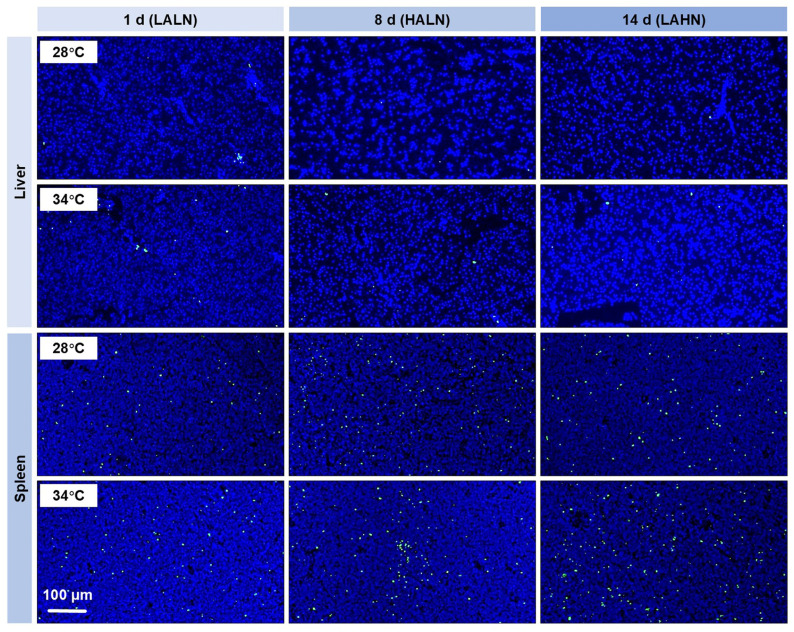
Representative photos of tissue sections assayed by TUNEL (terminal deoxynucleotidyl transferase dUTP Nick-End Labeling) (n = 3). Merged images of the green (apoptotic cells) and blue (nuclei) channels are shown. LALN, low ammonia and low nitrite levels; HALN, high ammonia and low nitrite levels; LAHN, low ammonia and high nitrite levels.

A low incidence of apoptotic cells was observed in the liver sections, with no significant difference in apoptosis between 28 °C and 34 °C throughout the experiment (Figure 4). In contrast, a higher incidence of apoptotic cells was observed in the spleen sections. Exposure to chronically accumulated ammonia (day 8) and nitrite (day 14) increased apoptotic intensity at both 28 °C and 34 °C (Figure 4, Figure S3B). Additionally, the spleen tissues of fish maintained at 34 °C showed a significantly higher apoptotic intensity compared to that at 28 °C on day 1 (*p* < 0.05, Figure S3B), suggesting an acute and quick effect of heat exposure.

### 3.5. Overall Changes in Gene Expression

**Figure 5:**
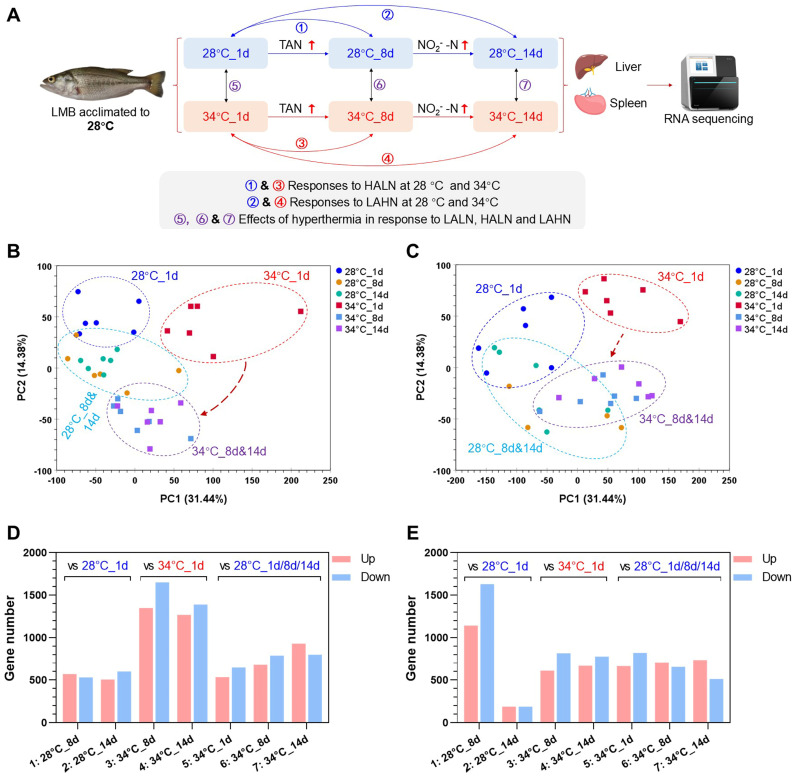
Experimental design and overall gene expression changes: (**A**) Experimental design. LMB juveniles were exposed to gradually increasing levels of ammonia (TAN) followed by high levels of nitrite (NO_2_^−^-N) at 28 °C and 34 °C. Gene expression in the liver and spleen was compared among different experimental groups to analyze the transcriptional responses to high levels of ammonia and nitrite, as well as the impact of hyperthermia on these responses. LALN, low ammonia and low nitrite levels (day 1); HALN, high ammonia and low nitrite levels (day 8); LAHN, low ammonia and high nitrite levels (day 14). (**B**,**C**) Principal component analysis results for gene abundance data in the liver (**B**) and spleen (**C**). (**D**,**E**) The number of differentially expressed genes (DEGs) in the liver (**D**) and spleen (**E**) under different conditions. The numbers preceding the experimental group names on the x-axis correspond to the sets of DEGs defined in (**A**). The arrows indicate the direction of sample location changes in the high-temperature group due to ammonia/nitrite accumulation.

The experimental design for RNA-seq analysis is illustrated in Figure 5A. The PCA results showed clear separation of liver samples at 28 °C_8 d and 28 °C_14 d from 28 °C_1 d, indicating the impact of ammonia and nitrite exposure. However, the samples at 28 °C_8 d and 28 °C_14 d could not be clearly separated. A similar pattern was observed for samples of 34°C, with a more pronounced separation between samples of 34 °C_1 d and 34 °C_8 d & 14 d, suggesting a combined effect of heat stress and ammonia and nitrite toxicities (Figure 5B). Comparable trends were seen in spleen samples (Figure 5C).

Heat exposure had a significant impact on the number of differentially expressed genes (DEGs). The liver of fish raised at 34 °C showed a higher number of DEGs on day 8 and day 14 compared to those raised at 28 °C, indicating a larger transcriptional response to both ammonia and nitrite (Figure 5D). Comparing gene expressions in the liver samples collected on the same days from fish raised under different temperatures also revealed a large number of DEGs (Figure 5D). In the spleen, fish raised at 34 °C had fewer DEGs on day 8 but more DEGs on day 14 compared to those raised at 28 °C (Figure 5E), suggesting that hyperthermia may decrease the transcriptional response to HALN but increase the response to LAHN in the spleen.

### 3.6. Biological Processes Affected by Hyperthermia and Ammonia/Nitrite Toxicity

**Figure 6:**
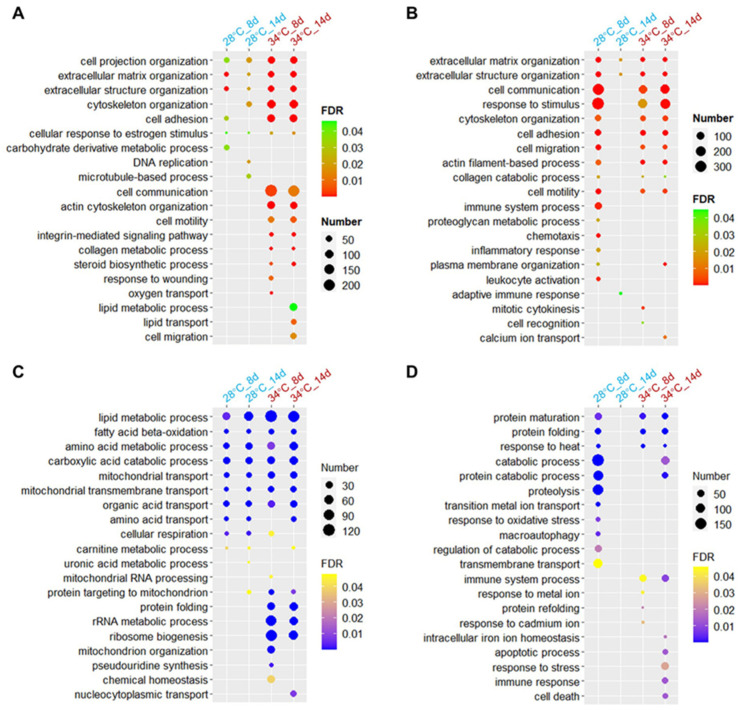
Gene ontology (biological process) enrichments for the differentially expressed genes (DEGs): (**A**,**B**) Upregulated genes in the liver (**A**) and spleen (**B**). (**C**,**D**) Downregulated genes in the liver (**C**) and spleen (**D**). The DEGs were identified by comparing gene expressions under the specified conditions with those of the corresponding tissues at 28 °C_1 d or 34 °C_1 d. Number, number of DEGs associated with the GO terms. FDR (false discovery rate), indicating the significance of the enrichments.

Gene ontology (GO) enrichment analysis was conducted on the DEGs to investigate the biological processes affected by chronic ammonia/nitrite toxicity at varying temperatures. In the liver of fish raised at 28 °C, upregulated genes on day 8 and day 14 were enriched in processes such as extracellular matrix and cytoskeleton organization, as well as cell adhesion. A greater number of genes associated with these processes were upregulated in the liver of fish raised at 34 °C, showing a higher level of enrichment (Figure 6A). Furthermore, genes upregulated at 34 °C were also enriched in terms such as cell communication and cell motility (Figure 6A).

The upregulated genes in the spleen at 28 °C_8 d showed an overrepresentation of multiple GO terms such as cell communication, response to stimulus, and cell adhesion. However, these terms were not overrepresented in the upregulated genes at 28 °C_14 d (Figure 6B), indicating a specific impact of HALN. On the other hand, these processes were found to be overrepresented by the upregulated genes at both 34 °C_8 d and 34 °C_14 d. The genes upregulated in the spleen at both temperatures were enriched in extracellular matrix organization. Exposure to 34 °C resulted in a more significant enrichment of upregulated genes in this process on day 14 (Figure 6B).

Accumulation of ammonia/nitrite led to the downregulation of genes involved in processes such as lipid metabolism, amino acid metabolism, and carboxylic acid catabolism in the liver at both temperatures, with more pronounced effects observed at 34 °C (Figure 6C). Additionally, exposure to both hyperthermia and ammonia/nitrite toxicity led to the downregulation of genes involved in processes such as protein targeting to mitochondria, protein folding, and rRNA metabolic process (Figure 6C).

Gene ontology terms enriched among downregulated genes in the spleen at 28 °C_8 d, such as protein maturation, protein folding, and catabolic process, were not enriched at 28 °C_14 d. However, these processes were overrepresented in genes downregulated at both 34 °C_8 d and 34 °C_14 d (Figure 6D). This pattern is consistent with the upregulated genes.

Together, these results suggest that hyperthermia exacerbated the impact of high levels of ammonia and nitrite on biological processes in the liver. In contrast, in the spleen, heat exposure mitigated the effects of ammonia (HALN) but intensified those of nitrite (LAHN).

### 3.7. Gene Modules Affected by Hyperthermia and Ammonia/Nitrite Toxicity

Weighted gene co-expression network analysis (WGCNA) was performed on the DEGs to identify gene modules and hub genes affected by hyperthermia in response to ammonia/nitrite toxicity. The DEGs of the liver and spleen were classified into 17 and 15 modules, respectively (Figure S4A,B). The correlation between gene modules and experimental parameters such as temperature, treatment duration, and levels of ammonia, nitrite, and nitrate, as well as phenotypic indices like SOD, CAT, and MDA levels in the liver were analyzed. Modules that exhibited significant correlations with temperature were highlighted in Figure S4C,D.

**Figure 7:**
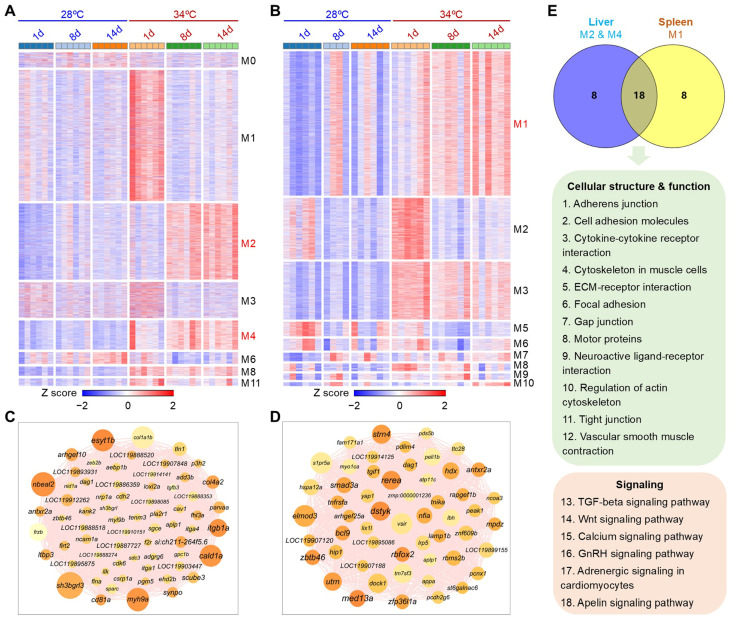
Gene modules affected by hyperthermia in response to ammonia/nitrite toxicity: (**A**,**B**) Heatmaps demonstrating the expression profiles of genes in selected gene modules in the liver (**A**) and spleen (**B**). These modules were identified through weighted gene co-expression network analysis (WGCNA) and were found to be significantly correlated with factors such as temperature; exposure time; and concentrations of TAN, nitrite, and nitrate (Figure S4). Different sampling days correspond to varying ammonia and nitrite levels: 1 d for low ammonia and low nitrite levels (LALN), 8 d for high ammonia and low nitrite levels (HALN), and 14 d for low ammonia and high nitrite levels (LAHN). (**C**,**D**) Hub genes of M2 (module #2) for the liver (**C**) and M1 (module #1) for the spleen (**D**). Larger node size and darker node color indicate a higher MCC score. (**E**) KEGG pathway enrichments for the genes of the indicated modules. The genes of M2 and M4 for the liver were combined for KEGG pathway enrichment analysis. The common pathways involved in cellular structure, function, and signaling are highlighted with green and red backgrounds, respectively.

The largest liver module, M1, showed transient induction at 34 °C on day 1, indicating an acute impact of heat exposure. Modules M2 and M4 were consistently upregulated across samples at 34 °C on day 8 and day 14 (Figure 7A), suggesting a synergistic effect of hyperthermia and ammonia/nitrite toxicity on gene expressions in these modules. M2, with more genes, was larger than M4. A network of hub genes in M2 is shown in Figure 7B, with genes like *nbeal2*, *esyt1b*, *sh3bgrl3*, *myh9a*, *cald1a*, and *itgb1a* identified as top markers for the combined effects of hyperthermia and ammonia/nitrite toxicity in the liver.

The largest spleen module (M1) showed upregulation at 34 °C on day 8 and day 14 (Figure 7C), suggesting a significant impact of hyperthermia and ammonia/nitrite toxicity on gene expression. Module M2 was transiently upregulated on day 1 at 34 °C, while module M3 consistently showed upregulation at 34 °C throughout the experiment, unaffected by ammonia/nitrite accumulation (Figure 7C). Representative hub genes of M1 for the spleen included *rerea*, *dstyk*, *strn4*, *med13a,* and *elmod3* (Figure 7D).

The genes in liver modules M2 and M4 were combined and subjected to KEGG pathways enrichment analysis along with the genes in spleen M1. The analysis revealed a significant overlap in pathway enrichments between the liver (M2 and M4) and spleen (M1) (Figure 7E), indicating similar effects of hyperthermia in both organs upon ammonia/nitrite accumulation. The common enrichments are mainly involved in structure functions such as cell adhesion, extracellular matrix and cytoskeleton, and pathways like TGF-beta and Wnt signaling (Figure 7E).

### 3.8. Gene Expressions Measured by qPCR

Liver and spleen samples from both experiments were analyzed using qPCR to assess gene expression. The study focused on genes involved in ECM–receptor interaction and TGF-beta signaling to differentiate the effects of heat exposure from the combined impacts of hyperthermia and ammonia/nitrite toxicity. These pathways were chosen as they are the typical ones affected by hyperthermia in the presence of ammonia/nitrite accumulation (Figure 7E).

**Figure 8:**
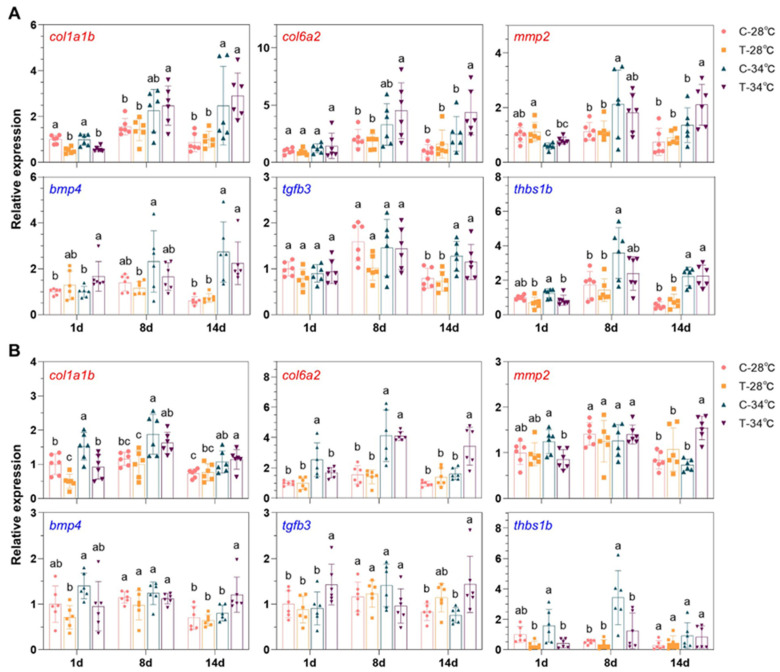
Relative expression of selected genes measured by qPCR: (**A**,**B**) Data for the liver (**A**) and spleen (**B**) samples. Gene expression under different conditions was normalized to the average expression at C-28 °C on day 1, which was set as the baseline value of 1. Genes related to ECM (extracellular matrix)–receptor interaction are shown in blue, while those associated with TGF-beta signaling are shown in red. Error bars represent standard deviation (n = 6). Different letters above the error bars indicate significant differences between the means of samples within the same group (Duncan multiple comparison test, *p* < 0.05). In the experimental groups, “C” denotes the control group without ammonia/nitrite accumulation, while “T” denotes the treatment group with ammonia/nitrite accumulation. Different sampling days correspond to varying ammonia and nitrite levels: 1 d for low ammonia and low nitrite levels (LALN), 8 d for high ammonia and low nitrite levels (HALN), and 14 d for low ammonia and high nitrite levels (LAHN).

The upregulation of *col1a1b* in both the liver and spleen due to heat exposure was not influenced by ammonia/nitrite toxicity (Figure 8A,B). However, the expressions of *col6a2* and *mmp2* in the liver and spleen of T-34 °C fish (34 °C, with ammonia/nitrite accumulation) were significantly higher compared to C-34 °C fish (34 °C, without ammonia/nitrite accumulation) (Figure 8A,B), indicating a synergistic effect of heat exposure and ammonia/nitrite toxicity on these genes involved in ECM–receptor interaction.

Genes involved in TGF-beta signaling were upregulated in the liver by heat exposure alone, as no significant difference was observed between the C-34 °C and T-34 °C samples on day 8 and day 14 (Figure 8A). However, in the spleen, the expressions of *bmp4* and *tgfb3* were significantly higher in the T-34 °C samples compared to the C-34 °C samples on day 14, suggesting a combined effect of hyperthermia and nitrite toxicity.

## 4. Discussion

Ammonia and nitrite are common pollutants in aquaculture systems, posing a significant threat to farmed aquatic animals [13,25]. These pollutants are produced through the microbial breakdown of residual feed, feces, and fish excretion, primarily from the catabolism of amino acids [16,23,25,64], with feed being the primary source. Elevated temperatures can increase the rate of ammonia excretion by fish and promote microbial growth [64]. However, the effects of high temperatures on the dynamics of ammonia and nitrite accumulation in newly established aquaculture systems, as well as the impacts of hyperthermia on the toxicity of these pollutants to fish, are not fully understood. Previous studies have mainly focused on the acute toxicity of high levels of nitrogenous compounds in the water [40,41], while the chronic toxicity of ammonia and nitrite at environmental levels remains to be explored. In this study, we developed a chronic exposure method for ammonia and nitrite, where nitrogen is introduced solely through feeding. Using this method, we investigated the influence of high temperatures on the accumulation of ammonia and nitrite, as well as the effects of hyperthermia on the toxicity of these pollutants in the liver and spleen of largemouth bass.

The higher temperature (34 °C) accelerated the accumulation of ammonia in the first half of the experiment and enhanced the accumulation of both nitrite and nitrate in the second half. In this study, higher total nitrogen (TN) concentrations in the water and an elevated feed conversion ratio (FCR) of the fish at 34 °C suggest that the fish assimilated less nitrogen from the feed at this temperature, resulting in increased nitrogen release into the water. Our findings also indicate a significant impact of temperature on the microorganisms involved in the conversion of ammonia to nitrite. Nitrification involves several steps: ammonia oxidation to hydroxylamine, hydroxylamine oxidation to nitric oxide and then to nitrite, and finally nitrite oxidation to nitrate [65]. Nitrogen-transforming microorganisms exhibit great metabolic versatility [66]. Recently, many novel microorganisms with unique metabolic capabilities have been discovered, such as comammox *Nitrospira*, which can oxidize both ammonia and nitrite to nitrate [66,67]. The rapid decrease in ammonia levels and the accumulation of nitrite in our system suggest a high abundance of microorganisms containing ammonia-oxidizing enzymes. However, the continuous accumulation of nitrite indicates a deficiency of microorganisms containing nitrite oxidoreductase (NXR), which oxidize nitrite to nitrate [68]. Further investigations focusing on the microbial composition at different temperatures and ammonia/nitrite concentrations can offer new insights into the effects of high temperatures on nitrogen cycling. Our data highlight the importance of introducing microorganisms capable of converting nitrite to nitrate, particularly for newly established aquaculture systems.

The toxicity of ammonia and nitrite to largemouth bass (LMB) has been previously studied. The 96 h LC50 value of ammonia for LMB at pH 7.8 and a temperature of 25 °C was estimated to be 33.24 mg/L [38]. Juvenile LMB exposed to high levels of ammonia (8.31 mg/L) for 28 days showed temporary disturbances in liver glycogen and protein levels, as well as plasma ion balance [38]. However, the fish were able to adapt to this level of ammonia by the end of the exposure period [38], indicating their ability to regulate ammonia toxicity. Exposure of LMB to 13 mg/L of ammonia at 26 ± 2 °C for 3 and 7 days resulted in severe liver damage [41]. Additionally, simultaneous exposure to ammonia (6.0 ± 0.5 mg/L) and hypoxia (1.2 ± 0.2 mg/L dissolved oxygen) at 24 ± 0.1 °C was found to have more harmful effects on LMB than individual stressors [69]. High concentrations of nitrite (400 or 600 mg/L) for a short duration (20 min) caused widespread tissue damage and apoptosis in LMB, with the spleen being the most affected organ [70,71]. These studies involved maintaining stable nitrogenous compound levels in the water throughout the experiments.

Our study is the first to investigate the toxicity of ammonia and nitrite that accumulate endogenously through feeding on LMB, with a focus on the effects of hyperthermia. The results indicate that successive exposure to gradually accumulated ammonia and nitrite resulted in oxidative stress, tissue damage, and apoptosis in the tissues of LMB. Moreover, more severe effects were observed in fish raised at 34 °C compared to 28 °C, particularly in the liver, suggesting further impairment of the metabolic and immunological functions. Chronic heat exposure alone can cause tissue damage in critical organs such as the liver and spleen of fish [12,45,46], so it is important to consider the toxicity of ammonia and nitrite during the summer months. Fish raised in enclosed systems or living in open water with the inflow of industrial and domestic wastewater containing high levels of ammonia and nitrite are at risk of increased toxicity when exposed to high temperatures. Global warming may exacerbate this issue by increasing the frequency and intensity of droughts [72], leading to unexpected insufficient water supply in regions with intensive aquaculture and making it challenging to remove ammonia and nitrite through water exchange. This may increase the risk of combined exposure to hyperthermia and high levels of ammonia and nitrite in farmed fish. Our findings from a simulated zero-water exchange system offer valuable insights for developing strategies to prevent ammonia and nitrite accumulation and enhance fish health in the face of combined hyperthermia and ammonia/nitrite toxicity.

Exposure to ammonia/nitrite had a significant impact on gene expression, particularly upregulating genes related to extracellular matrix (ECM) and cytoskeleton organization, with a more pronounced effect observed in fish raised at 34 °C. Previous studies in shrimps have also shown that cytoskeleton remodeling plays a role in the response to ammonia stress [73,74]. The cytoskeleton is involved in cell volume regulation, and its organization can be affected by perturbations to cell volume [75]. The ECM, which is a crucial cellular microenvironment, also regulates cell volume [76]. The upregulation of genes involved in the cytoskeleton and ECM organization may be linked to increased cell size, especially in the liver of LMB exposed to ammonia/nitrite toxicity. Previous research has shown that ammonia can lead to the enlargement of hepatocytes in LMB and other species [36,41,77]. In the gills of fish exposed to ammonia toxicity, enlargement of the interlamellar cell mass (ILCM) has been observed, with the highly ammonia-resistant goldfish (*Carassius auratus*) showing the most significant changes [78]. This response is considered a survival strategy in an ammonia-rich environment [78]. Despite being exposed to hyperthermia and ammonia/nitrite toxicity for 14 days, the experimental fish appeared healthy, suggesting they may have adapted to the conditions. Therefore, the upregulation of genes involved in these pathways may serve as both a stress response and an adaptive strategy to high ammonia/nitrite environments.

Exposure to hyperthermia, ammonia, and nitrite can alter gene expression in fish tissues [6,12,30,45]. The ECM genes *col6a2* and *mmp2* (in both the liver and spleen) and TGFβ signaling genes *bmp4* and *tgfb3* (in the spleen) are reliable indicators of the combined effects of hyperthermia and high nitrite levels on day 14. However, no combined effects were observed between hyperthermia and high ammonia levels on day 8. A previous study has shown that nitrite is more toxic to the head kidney of silver carp (*Hypophthalmichthys molitrix*) than ammonia [35]. The longer exposure time and higher peak nitrite levels in our system may explain the differential effects on gene expression. In conclusion, the effects of single stressors and combinations of multiple stressors depend on exposure time and tissue type.

## 5. Conclusions

A high temperature of 34 °C led to increased accumulation of ammonia, nitrite, nitrate, and total nitrogen, as well as accelerated conversion of ammonia to nitrite in a zero-water exchange system for largemouth bass with normal feeding. The fish experienced initial exposure to high levels of ammonia followed by high levels of nitrite due to the dynamic accumulation and transformation of ammonia. Successive exposure to ammonia and nitrite caused oxidative stress in the liver, with more pronounced effects observed in fish raised at 34 °C compared to 28 °C. Pathological changes in the liver and spleen were evident following exposure to ammonia and nitrite, with more severe impacts observed in fish at 34 °C. Increased apoptosis was only detected in the spleen, with hyperthermia showing no significant influence. Hyperthermia also affected gene expression in the liver and spleen in response to ammonia and nitrite exposure. Genes upregulated by ammonia and nitrite exposure were primarily involved in the cytoskeleton and extracellular matrix organization processes, with more differentially expressed genes involved in these processes identified in fish raised at 34 °C. Gene modules regulated by hyperthermia in response to ammonia and nitrite exposure in the liver and spleen were also identified. Furthermore, a combined effect of hyperthermia and nitrite exposure was observed in the expression of genes involved in ECM (extracellular matrix)–receptor interaction and TGFβ signaling, specifically *col6a2*, *mmp2*, *bmp4,* and *tgfb3*.

## Figures and Tables

**Table 1:** Concentrations of total ammonia, unionized ammonia, and nitrite on the sampling days in both experiments.

Pollutant	Time (Days)	Experiment #1		Experiment #2	
		28 °C	34 °C	28 °C	34 °C
Ammonia (TAN, mg/L)	1	0.45 ± 0.06 ^b^	0.68 ± 0.04 ^b,^**	0.53 ± 0.08 ^a^	0.16 ± 0.01 ^a,^*
	8	4.93 ± 0.44 ^a^	5.95 ± 0.83 ^a^	0.02 ± 0.02 ^b^	0.03 ± 0.02 ^b^
	14	0.10 ± 0.01 ^b^	0.15 ± 0.09 ^b^	0.01 ± 0.02 ^b^	0.02 ± 0.02 ^b^
Unionized Ammonia (NH_3_-N, mg/L)	1	0.02± 0.00 ^b^	0.03 ± 0.00 ^b,^**	0.02 ± 0.00	0.02 ± 0.01
	8	0.24 ± 0.02 ^a^	0.36 ± 0.06 ^a,^*	0.00 ± 0.00	0.00 ± 0.00
	14	0.01 ± 0.00 ^b^	0.01 ± 0.01 ^b^	0.00 ± 0.00	0.00 ± 0.00
Nitrite (NO_2_^−^-N, mg/L)	1	0.00 ± 0.00 ^b^	0.00 ± 0.00 ^c^	0.01 ± 0.00	0.01 ± 0.00
	8	0.56 ± 0.35 ^b^	1.50 ± 1.08 ^b^	0.04 ± 0.00	0.00 ± 0.00
	14	10.83 ± 0.42 ^a^	12.83 ± 0.21 ^a,^**	0.00 ± 0.00	0.00 ± 0.00

Different superscripts in the same column indicate significant differences among samples of the same experimental group (*p* < 0.05, Duncan’s multiple comparison). The significant difference between the experimental groups: *, *p* < 0.05 and **, *p* < 0.01 (Independent Samples *t*-test).

**Table 2:** Growth indices and feed conversion ratio of the experimental fish.

Item	28 °C	34 °C
Survival (%)	100.00 ± 0.00	94.87 ± 5.88
Initial weight (g)	632.00 ± 2.00	634.33 ± 1.46
Average initial weight (g)	21.07 ± 0.07	21.15 ± 0.05
Final weight (g)	523.33 ± 11.55	490.00 ± 10.00 *
Average final weight (g)	23.79 ± 0.53	22.27 ± 0.46 *
Feeding amount (g)	140.95 ± 2.14	141.69 ± 1.25
Average weight gain rate (WGR, %)	12.91 ± 2.19	5.33 ± 2.01 *
Average special growth rate (SGR, %)	0.87 ± 0.14	0.37 ± 0.14 *
Feed conversion ratio (FCR)	1.51 ± 0.11	2.98 ± 0.65 *

Data from Experiment #1 is displayed. The significant difference between the experimental groups: *, *p* < 0.05 (independent samples *t*-test).

## Data Availability

The article’s material includes the original contributions presented in this study. Further inquiries can be directed to the corresponding author.

## Supplementary Materials

The following supporting information can be downloaded at: https://www.mdpi.com/article/10.3390/antiox14040495/s1, Figure S1. Daily water parameters during Experiment #2: (A) Temperature. (B) Dissolved oxygen (DO). (C) Total ammonia nitrogen (TAN). (D) Nitrite (NO_2_^−^-N). The error bars represent standard error (n = 3). *, *p* < 0.05 (Independent samples *t*-test). Figure S2. Identification of internal reference genes for qPCR data normalization. The expression of the genes was analyzed using a qPCR assay, and the data were further analyzed with Normfinder. Figure S3. Density of melano-macrophage (MM) (A) and fluorescence intensity for TUNEL signal in the spleen tissue sections (B). Figure S4. Identification of gene modules affected by hyperthermia in response to high levels of ammonia and nitrite: (A and B) Heatmaps displaying the expression profiles of identified gene modules in the liver (A) and spleen (B) under various conditions. (C and D) Correlation of gene modules with experimental factors. The gene modules significantly correlated with temperature are highlighted in red. Table S1. Information for the primers used for qPCR assay.

## Abbreviations

The following abbreviations are used in this manuscript:

CAT	Catalase
DEGs	Differentially expressed genes
DO	Dissolved oxygen
ECM	Extracellular matrix
FCR	Feed conversion ratio
GO	Gene ontology
HALN	High ammonia and low nitrite
H&E	Hematoxylin and eosin
ILCM	Interlamellar cell mass
KEGG	Kyoto encyclopedia of genes and genomes
LAHN	Low ammonia and high nitrite
LALN	Low ammonia and low nitrite
LMB	Largemouth bass
MDA	Malondialdehyde
NH_3_	Nonionic ammonia
NH_4_^+^	Ionic ammonia
NO_3_-N	Nitrate
NO_2_-N	Nitrite
NXR	Nitrite oxidoreductase
PCA	Principal component analysis
PFA	Paraformaldehyde
SD	Standard deviation
SGR	Specific growth rate
SOD	Superoxide dismutase
SRA	Sequence read Archive
TAN	Total ammonia–nitrogen
TDT	Terminal deoxynucleotidy1 transferase
TN	Total nitrogen
TPM	Transcripts per million
WGCNA	Weighted gene co-expression network analysis
WGR	Weight gain rate
